# Platelet lysate induces chondrogenic differentiation of umbilical cord‐derived mesenchymal stem cells by regulating the lncRNA H19/miR‐29b‐3p/SOX9 axis

**DOI:** 10.1002/2211-5463.13002

**Published:** 2020-11-06

**Authors:** Boran Cao, Xin Dai

**Affiliations:** ^1^ Department of Orthopedics The First Affiliated Hospital of Harbin Medical University Harbin China; ^2^ Department of Oncology The First Affiliated Hospital of Harbin Medical University Harbin China

**Keywords:** chondrogenic differentiation, H19, mesenchymal stem cells, miR‐29b‐3p, platelet lysate, SOX9

## Abstract

Platelet lysate (PL) has been shown to induce chondrogenic differentiation of human umbilical cord‐derived mesenchymal stem cells (hUCMSCs). However, the underlying mechanism is still not clear. The aim of this study was to investigate whether long noncoding RNA H19 is involved in this process. hUCMSCs were isolated, identified and cultured in 5% PL‐supplemented chondrogenic differentiation medium. Chondrogenic differentiation was assessed by Alcian blue staining. The expressions of H19, miR‐29b‐3p, SRY‐related high‐mobility‐group box 9 (SOX9), collagen II and aggrecan were determined by quantitative real‐time PCR and western blot. The interaction between miR‐29b‐3p and H19 or SOX9 was analyzed by luciferase reporter assay. During PL‐induced chondrogenic differentiation of hUCMSCs, expressions of H19 and SOX9 were increased, whereas miR‐29b‐3p expression was decreased. H19 overexpression promoted, whereas H19 silencing attenuated the PL‐induced chondrogenic differentiation of hUCMSCs. SOX9 was identified as a direct target of miR‐29b‐3p, and H19 was observed to act as a sponge of miR‐29b‐3p to up‐regulate SOX9 expression. The chondrogenic differentiation‐promoting effect of H19 overexpression was negated when miR‐29b‐3p expression was up‐regulated by Lenti‐miR‐29b‐3p infection. In conclusion, PL induced chondrogenic differentiation of hUCMSCs by regulating the H19/miR‐29b‐3p/SOX9 axis.

## Graphical abstract

H19 expression is induced in platelet lysate‐stimulated human umbilical cord‐derived mesenchymal stem cells (hUCMSCs), and H19 acts as a sponge of miR‐29b‐3p to up‐regulate the expression of the master chondrocyte transcription factor SRY‐related high‐mobility‐group box 9, thereby promoting platelet lysate‐induced chondrogenic differentiation of hUCMSCs.

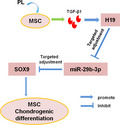

**Abbreviations**
hUCMSC: human umbilical cord‐derived mesenchymal stem cell
lncRNA: long noncoding RNA
MSC: mesenchymal stem cell
Mut: mutated
NC: negative control
OA: osteoarthritis
PL: platelet lysate
PRP: platelet‐rich plasma
qRT‐PCR: quantitative real‐time PCR
ROCR: regulator of chondrogenesis RNA
SOX9: SRY‐related high‐mobility‐group box 9
TGF‐β: transforming growth factor β
WT: wild‐type

Osteoarthritis (OA) is one of the major joint diseases worldwide mainly characterized by articular cartilage degeneration [1]. Articular cartilage is an important component of the synovial joints and lacks the capacity for self‐regeneration [2]. Mesenchymal stem cells (MSCs) have emerged as a relevant cell source for cartilage repair in recent years [3, 4]. Zheng *et al*. [5] observed that human chondrocytes could be successfully induced by coculture of human umbilical cord‐derived mesenchymal stem cells (hUCMSCs) with rabbit chondrocytes.

Platelet‐rich plasma (PRP), a blood extraction product widely used in clinical practice, can not only enhance tissue healing ability and promote tissue cell proliferation and differentiation but also greatly accelerates the healing process of tissue injuries [6, 7, 8]. Platelet lysate (PL), a purified product of PRP after lysis [9], contains high levels of growth factors important for MSC proliferation and differentiation, such as transforming growth factor β (TGF‐β), fibroblast growth factor and platelet‐derived growth factor [10]. Among these, TGF‐β is critically important to induce chondrogenic differentiation of MSCs [11]. A previous study demonstrated that PL induced chondrogenic differentiation of hUCMSCs, concomitant with elevated expression of SRY‐related high‐mobility‐group box 9 (SOX9), a cartilage‐specific transcription factor that plays crucial roles in chondrocyte differentiation [12]. However, the underlying mechanism is still not clear.

Long noncoding RNAs (lncRNAs) are non‐protein‐coding transcripts longer than 200 nucleotides and play important regulatory roles in various physiological and disease processes [13]. Recent studies have suggested that lncRNA plays a critical role in chondrogenic differentiation of MSCs. For example, Barter *et al*. [14] reported that lncRNA regulator of chondrogenesis RNA (ROCR) induced chondrogenic differentiation of MSCs by up‐regulating SOX9 expression. H19, a kind of lncRNA, has been shown to promote the osteogenic differentiation of MSCs [15, 16] but inhibit the differentiation MSCs into adipocytes [17]. Existing evidence has demonstrated that H19 expression can be induced by TGF‐β that presents in PL [10, 18], suggesting that H19 might be involved in the PL‐induced chondrogenic differentiation of hUCMSCs. In recent years, many studies have indicated that H19 is implicated in tenogenic differentiation [19], tumor cell epithelial–mesenchymal transition [20, 21] and myocardial ischemia–reperfusion injury [22] by functioning as a miR‐29b‐3p sponge via competing endogenous RNA activity. It should be noted that miR‐29b‐3p is predicted to bind to the 3′‐UTR of the master chondrocyte transcription factor SOX9 by bioinformatics software (http://starbase.sysu.edu.cn/). Thus, we hypothesized that H19 might be involved in PL‐induced chondrogenic differentiation of hUCMSCs by regulating the miR‐29b‐3p/SOX9 axis.

## Materials and methods

### Preparation of human PLs

Human PLs were prepared from PRP obtained from five donors as previously described [12]. In brief, 20 mL of peripheral blood was collected from each healthy volunteer and pooled. PRP was isolated from peripheral blood and then subjected to three cycles of freeze–thaw (frozen at −80 °C and thawed at 37 °C) to obtain PL. PL was centrifuged at 15 000 ***g*** for 20 min at 4 °C, filtered through 0.22‐μm filters and stored at −20 °C until use. Written informed consent was obtained from all volunteers. This experiment was approved by the Ethics Committee of First Affiliated Hospital of Harbin Medical University. The study methodologies conformed to the standards set by the Declaration of Helsinki.

### Isolation of hUCMSCs

hUCMSCs were isolated from umbilical cords of five cesarean‐delivered full‐term neonates using the explant method [12]. Informed consent was obtained from the parent/legal guardian. In brief, umbilical cords were cut into smaller pieces of ~5 × 5 cm, transferred to a 25‐cm^2^ flask and maintained in Dulbecco’s modified Eagle’s medium (Gibco, Thermo Fisher Scientific, Inc., Waltham, MA, USA) supplemented with 5% PL, 100 μg·mL^−1^ streptomycin and 100 U·mL^−1^ penicillin at 37 °C in a humidified atmosphere containing 5% CO_2_. The medium was replaced for the first time after 7 days of culture and thereafter every 3–4 days. The umbilical cord segments were removed on the 14th day, and adherent cells reaching 75–85% confluence were detached by 0.05% trypsin/EDTA and further cultivated until the third passage. hUCMSCs at passage 2 were used in the following experiments.

### Immunophenotypic characterization of hUCMSCs

hUCMSCs at passage 2 were characterized by flow cytometry. In brief, cells were washed twice with PBS, digested by 0.05% trypsin and suspended at a concentration of 5 × 10^5^ cells per tube. Cells were then stained at 4 °C with the following antibodies: CD34‐phycoerythrin (Invitrogen, Carlsbad, CA, USA), CD45‐FITC (Abbexa, Cambridge, UK), CD44‐FITC (Miltenyi Biotec, San Diego, CA, USA) and CD105‐phycoerythrin (Invitrogen). After 30 min of incubation in the dark, cells were washed with PBS and analyzed using flow cytometry (Beckman Coulter, Brea, CA, USA).

### Chondrogenic differentiation

hUCMSCs at passage 2 (1 × 10^6^ cells) were cultured in a chondrogenic differentiation medium supplemented with Dulbecco’s modified Eagle’s medium, 1 × insulin‐transferrin‐selenium, 50 mg·L^−1^ ascorbic acid, 100 nm dexamethasone and 5% PL for 21 days. The culture medium was replaced twice a week.

### Cell infection and transfection

The miR‐29b‐3p precursor‐expressing lentiviral particles (Lenti‐miR‐29b‐3p) and control lentivirus particles (Lenti‐miR‐NC), H19‐expressing lentivirus (Lenti‐H19) and control lentivirus particles (Lenti‐NC), and sh‐H19‐expressing lentivirus (Lenti‐sh‐H19) and sh‐NC‐expressing lentivirus (Lenti‐shRNA) were purchased from Hanbio (Shanghai, China). The miR‐29b‐3p mimic, mimic NC, miR‐29b‐3p inhibitor and inhibitor NC were purchased from GenePharma (Shanghai, China). The hUCMSCs at 70–80% confluence were infected with these lentiviruses or transfected with the mimics and/or inhibitors using Lipofectamine 3000 (Invitrogen) according to the manufacturer’s instructions. Cells were harvested 48 h later and subjected to further experiments.

### Alcian blue staining

After infection with the indicated lentiviruses, hUCMSCs were cultured in a chondrogenic differentiation medium for 21 days as described earlier. Then chondrogenic differentiation was assessed by Alcian blue staining. In brief, the pellets were fixed, dehydrated, embedded in paraffin and sectioned at 5 μm thickness. For detection of proteoglycan and mucopolysaccharides, sections were deparaffinized, rehydrated and then stained with 1% Alcian blue (Sigma‐Aldrich, St. Louis, MO, USA) for 30 min.

### Luciferase reporter assay

The fragments of H19 containing the predicted wild‐type (WT) or mutated (Mut) miR‐29b‐3p binding sites were amplified by PCR and inserted into a pGL3‐luciferase vector (Promega, Madison, WI, USA), generating pGL3‐H19 WT/pGL3‐H19 Mut luciferase constructs. Similarly, the fragments of SOX9 3′‐UTR containing the predicted WT or Mut miR‐29b‐3p binding sites were amplified by PCR and inserted into a pGL3‐luciferase vector, generating pGL3‐SOX9 WT or pGL3‐SOX9 Mut constructs. The primers were as follows: H19 WT construct primer forward, 5′‐ ACTGGAATTCTTACTTCCTCCACGGAGTCG‐3′; H19 WT construct primer reverse, 5′‐ACTGCTCGAGTGTTCCGATGGTGTCTTTGA‐3′; SOX9 WT 3′‐UTR construct primer forward, 5′‐ACTGGAATTCTCAGGCTTTGCGATTTAAGG‐3′; SOX9 WT 3′‐UTR construct primer reverse, 5′‐ ACTGCTCGAGAGGCAGGAGGAAATGCACTA‐3′. HEK 293T cells at 75–85% confluence were cotransfected with these constructs, miR‐29b‐3p mimic/mimic NC and pRL‐TK (a plasmid expressing Renilla luciferase) using Lipofectamine 2000 (Invitrogen). The luciferase activities 24 h after transfection were analyzed using a luciferase reporter assay system (Promega).

### Quantitative real‐time PCR

Total RNA was extracted from cultured hUCMSCs using TRIzol Reagent (Invitrogen) and subjected to reverse transcription reactions using a PrimeScript RT Reagent Kit (Takara, Dalian, China). The expression levels of miR‐29b‐3p were determined using the miRNA quantitative real‐time PCR (qRT‐PCR) kit (GeneCopoeia, Rockville, MD, USA), whereas the expression levels of H19 and SOX9 were detected using the SYBR premix (Takara) on Applied Biosystems 7500 PCR system. Relative expression levels of candidate genes were calculated by the $2^{‐\Delta \Delta C_{t}}$ method and normalized to U6 (internal control for miR‐29b‐3p) or GAPDH (internal control for H19 and SOX9). The primers were as follows: H19 forward, 5′‐TCTGAGAGATTCAAAGCCTCCAC‐3′; H19 reverse, 5′‐GTCTCCACAACTCCAACCAGTG‐3′; SOX9 forward, 5′‐ AGGAAGCTCGCGGACCAGTAC‐3′; SOX9 forward, 5′‐ GGTGGTCCTTCTTGTGCTGCAC‐3′; miR‐29b‐3p forward, 5′‐ACACTCCAGCTGGGTAGCACCATTTGAAATC‐3′; miR‐29b‐3p reverse, 5′‐ TGGTGTCGTGGAGTCG‐3′; GAPDH forward, 5′‐ GTCTCCTCTGACTTCAACAGCG‐3′; GAPDH reverse, 5′‐ ACCACCCTGTTGCTGTAGCCAA‐3′; U6 forward, 5′‐ TGCGGGTGCTCGCTTCGGCAGC‐3′; U6 reverse, 5′‐GTGCAGGGTCCGAGGT‐3′.

### Western blot

The whole‐cell lysates were extracted from hUCMSCs using the radioimmunoprecipitation assay lysis buffer (Beyotime, Haimen, China). Then the protein samples were loaded and separated with 10% SDS/PAGE gels and transferred to poly(vinylidene difluoride) membrane. After being blocked with 5% nonfat milk, the membranes were incubated with primary antibodies (1 : 1000) at 4 °C overnight, followed by the horseradish peroxidase‐conjugated secondary antibodies (1 : 2000; Abcam, Cambridge, MA, USA). Blots were examined by an ECL Detection kit (Pierce Biotechnology, Rockford, IL, USA). The primary antibodies used were as follows: anti‐collagen II (#ab239007), anti‐aggrecan (#ab36861), anti‐SOX9 (#ab185230) and anti‐α‐tubulin (#ab7291) (1 : 1000 dilution; all from Abcam).

### Statistical analysis

All statistical analyses were performed using spss 22.0 (SPSS, Inc., Chicago, IL, USA). Statistical significance was analyzed using the Student's *t*‐test between two groups and one‐way ANOVA among three or more groups. A *P* value <0.05 was considered statistically significant.

## Results

### H19 expression was increased during PL‐induced chondrogenic differentiation of hUCMSCs

**Fig. 1:**
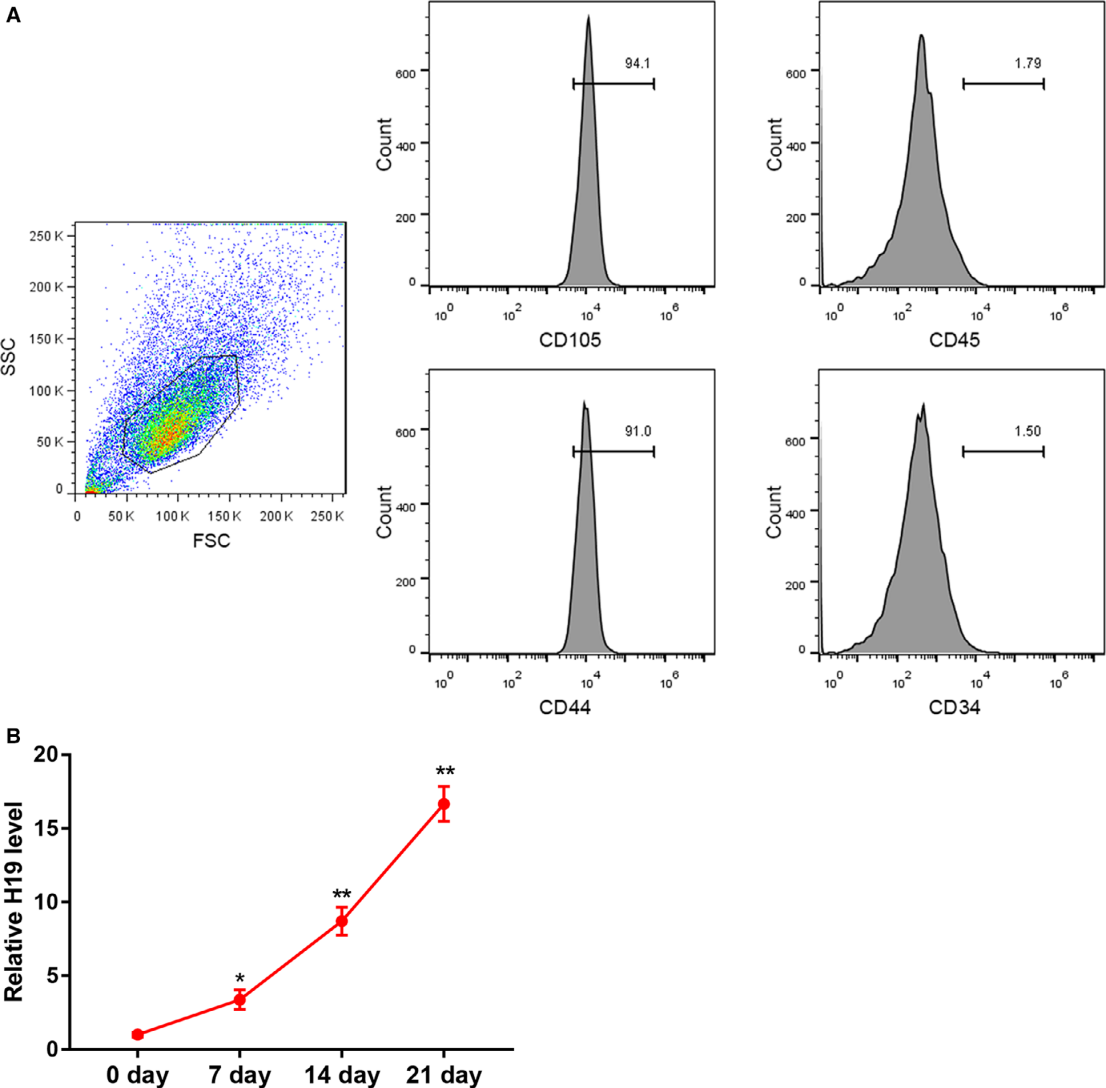
H19 expression was increased during PL‐induced chondrogenic differentiation of hUCMSCs. (A) hUCMSCs at passage 2 were characterized by flow cytometry. Forward scatter (FSC) and side scatter (SSC) distribution of gated cells (left). The cells were positive for CD105 and CD44, but negative for CD45 and CD34 (right). (B) qRT‐PCR analysis of H19 expression in hUCMSCs cultured in 5% PL‐supplemented chondrogenic differentiation medium for 0, 7, 14 and 21 days. *P < 0.05, **P < 0.01, vs. 0 day (ANOVA). The quantitative statistics were presented as the mean ± standard deviation (*n* = 3).

hUCMSCs were isolated from umbilical cords of cesarean‐delivered full‐term neonates and identified by flow cytometry. Data revealed that the obtained hUCMSCs were positive for CD105 and CD44, but negative for CD45 and CD34 (Fig. 1A), confirming that the cells were MSCs. hUCMSCs were then cultured in 5% PL‐supplemented chondrogenic differentiation medium for 0, 7, 14 and 21 days. The results from qRT‐PCR analysis demonstrated significant up‐regulation of H19 expression during PL‐induced chondrogenic differentiation of hUCMSCs (Fig. 1B).

### H19 promoted PL‐induced chondrogenic differentiation of hUCMSCs

**Fig. 2:**
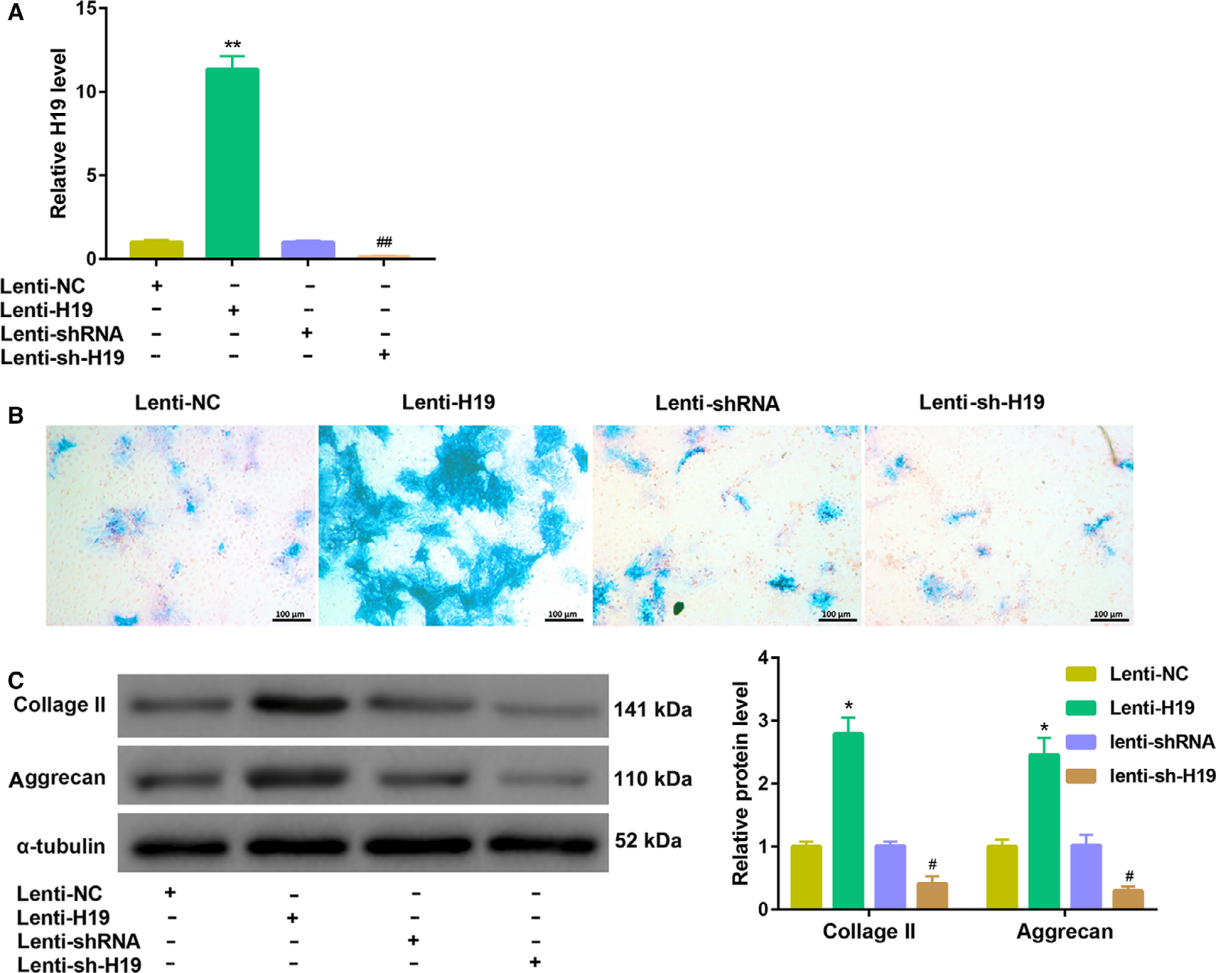
Effects of H19 overexpression and silencing on PL‐induced chondrogenic differentiation of hUCMSCs. (A) qRT‐PCR analysis of H19 expression in hUCMSCs at 48 h after infection with Lenti‐NC, Lenti‐H19, Lenti‐shRNA and Lenti‐sh‐H19. (B, C) The infected hUCMSCs as described in (A) were cultured in 5% PL‐supplemented chondrogenic differentiation medium for 21 days. (B) Chondrogenic differentiation was assessed by Alcian blue staining (scale bar: 100 μm). (C) The protein levels of collagen II and aggrecan in hUCMSCs were examined by western blot. *P < 0.05, **P < 0.01, vs. Lenti‐NC; ^#^ *P* < 0.05, ^##^ *P* < 0.01, vs. Lenti‐shRNA (Student's *t*‐test). The quantitative statistics were presented as the mean ± standard deviation (*n* = 3).

To determine the role of H19 in PL‐induced chondrogenic differentiation of hUCMSCs, we overexpressed and silenced H19 in hUCMSCs, and then cultured them in 5% PL‐supplemented chondrogenic differentiation medium for 21 days. The overexpression and silencing efficiencies of H19 were confirmed by qRT‐PCR analysis (Figs 2A and S1A). Importantly, Alcian blue staining demonstrated that H19 overexpression led to increased production of proteoglycan in the differentiated cells, whereas H19 silencing yielded the opposite results (Fig. 2B). Furthermore, the protein levels of chondrogenic markers (collagen II and aggrecan) in the differentiated cells were significantly up‐regulated by H19 overexpression but down‐regulated by H19 silencing (Fig. 2C). These findings indicated that H19 promoted PL‐induced chondrogenic differentiation of hUCMSCs.

### H19 acted as a sponge of miR‐29b‐3p to up‐regulate SOX9 expression

**Fig. 3:**
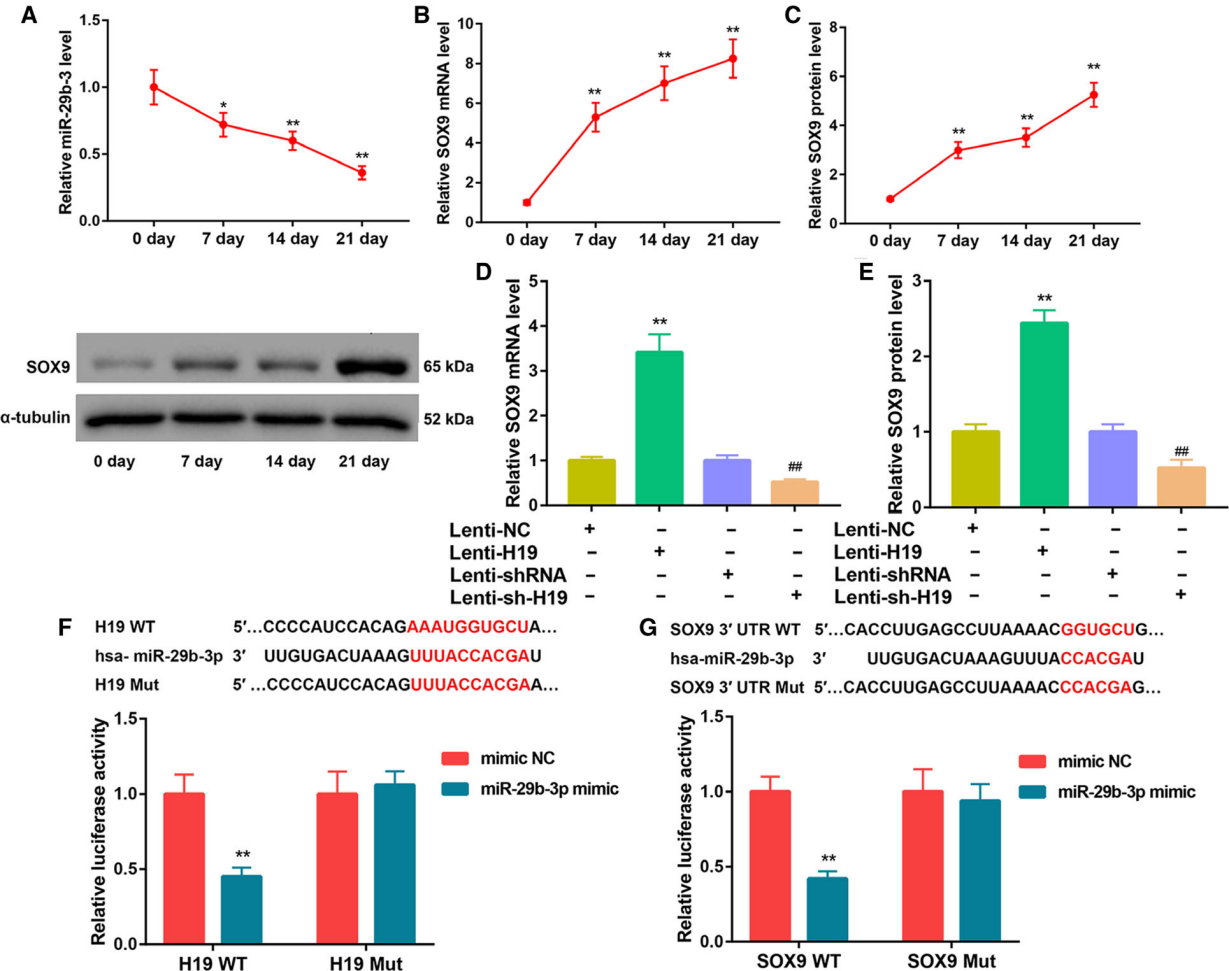
Interaction between miR‐29b‐3p and H19 or SOX9. qRT‐PCR analysis of (A) miR‐29b‐3p expression and (B) SOX9 mRNA level, and (C) western blot analysis of SOX9 protein level in hUCMSCs cultured in 5% PL‐supplemented chondrogenic differentiation medium for 0, 7, 14 and 21 days. *P < 0.05, **P < 0.01, vs. 0 day (Student's *t*‐test). (D, E) qRT‐PCR analysis of SOX9 mRNA level (D) and western blot analysis of SOX9 protein level (E) in hUCMSCs infected with Lenti‐NC, Lenti‐H19, Lenti‐shRNA and Lenti‐sh‐H19. **P < 0.01, vs. Lenti‐NC; ^##^ *P* < 0.01, vs. Lenti‐shRNA (Student's *t*‐test). (F) The WT (H19 WT) and mutation (H19 Mut) of binding sites between H19 and miR‐29b‐3p. Results of luciferase activity assay verified the direct binding between H19 and miR‐29b‐3p. (G) The WT (SOX9 WT) and mutation (SOX9 Mut) of binding sites between SOX9 and miR‐29b‐3p. Results of luciferase activity assay verified the direct binding between SOX9 3′‐UTR and miR‐29b‐3p. **P < 0.01, vs. mimic NC (Student's *t*‐test). The quantitative statistics were presented as the mean ± standard deviation (*n* = 3).

**Fig. 4:**
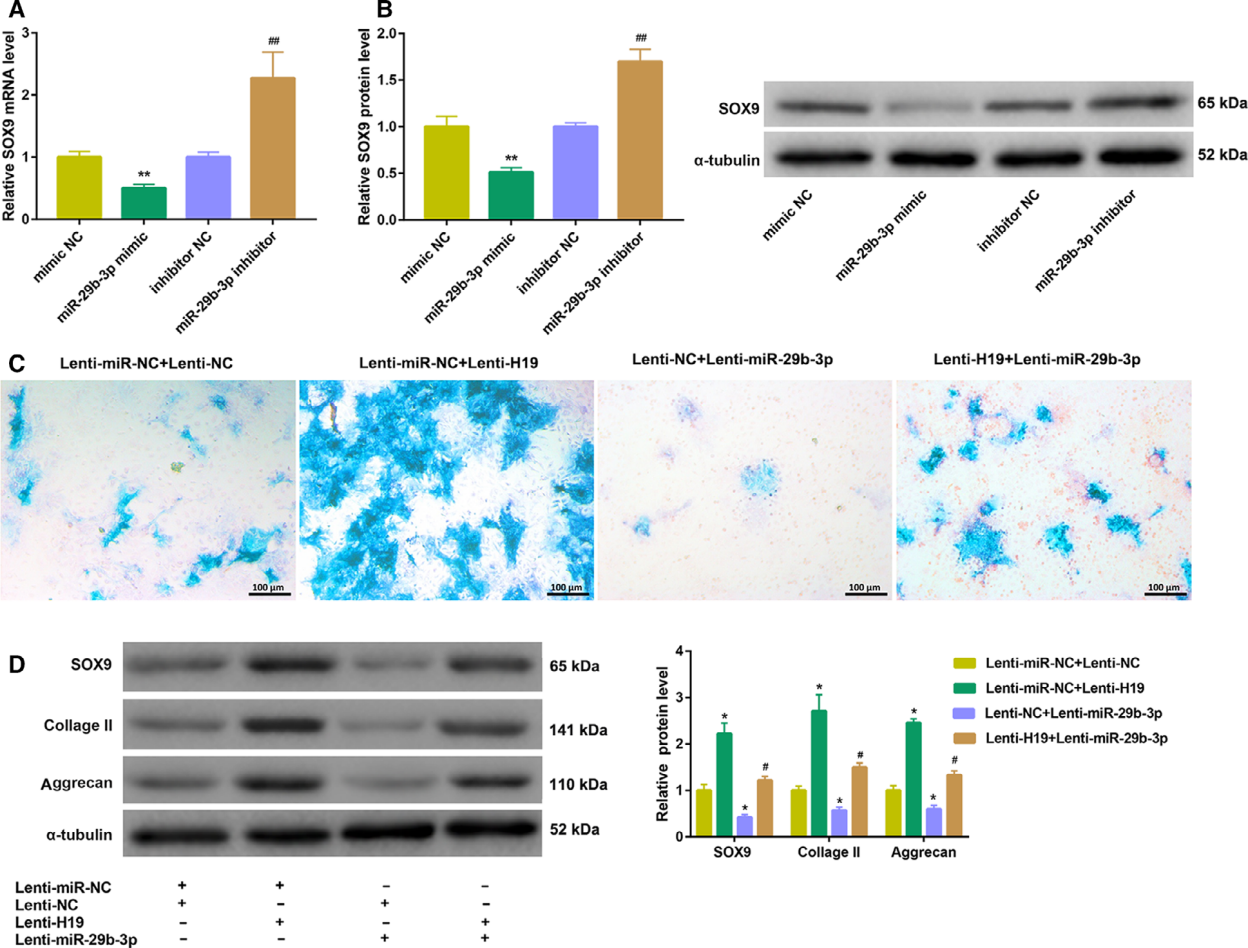
H19 promoted PL‐induced chondrogenic differentiation of hUCMSCs by regulating the miR‐29b‐3p/SOX9 axis. (A, B) qRT‐PCR analysis of SOX9 mRNA level (A) and western blot analysis of SOX9 protein level (B) in hUCMSCs transfected with miR‐29b‐3p mimic, miR‐29b‐3p inhibitor or corresponding controls. **P < 0.01, vs. mimic NC; ^##^ *P* < 0.01, vs. inhibitor NC (Student's *t*‐test). (C, D) hUCMSCs were coinfected with Lenti‐NC/Lenti‐H19 and Lenti‐miR‐NC/Lenti‐miR‐29b‐3p, and then cultured in 5% PL‐supplemented chondrogenic differentiation medium for 21 days. (C) Chondrogenic differentiation was assessed by Alcian blue staining (scale bars: 100 μm). (D) The protein levels of SOX9, collagen II and aggrecan in hUCMSCs were examined by western blot. *P < 0.05, vs. Lenti‐miR‐NC + Lenti‐NC; ^#^ *P* < 0.05, vs. Lenti‐miR‐NC + Lenti‐H19 (ANOVA). The quantitative statistics were presented as the mean ± standard deviation (*n* = 3).

Next, we explored the molecular mechanism by which H19 promotes PL‐induced chondrogenic differentiation of hUCMSCs. The results from qRT‐PCR analysis revealed that miR‐29b‐3p expression was notably down‐regulated, whereas SOX9 mRNA and protein levels were significantly up‐regulated during PL‐induced chondrogenic differentiation of hUCMSCs (Fig. 3A–C). Furthermore, H19 overexpression led to a marked increase in SOX9 mRNA and protein levels. In contrast, H19 silencing significantly down‐regulated SOX9 expression (Fig. 3D–E). In addition, the luciferase activity was significantly decreased in cells cotransfected with H19 WT reporter and miR‐29b‐3p mimic (Fig. 3F), indicating that H19 directly interacted with miR‐29b‐3p. We also observed decreased luciferase activity in cells cotransfected with SOX9 WT reporter and miR‐29b‐3p mimic (Fig. 3G), suggesting that 3′‐UTR of SOX9 was directly targeted by miR‐29b‐3p. Moreover, transfection with miR‐29b‐3p mimic resulted in a significant decrease in mRNA and protein levels of SOX9. On the contrary, inhibition of miR‐29b‐3p noticeably increased SOX9 expression (Fig. 4A,B). Together, these results manifested that H19 might act as a sponge of miR‐29b‐3p to up‐regulate SOX9 expression.

### H19 promoted PL‐induced chondrogenic differentiation of hUCMSCs by regulating the miR‐29b‐3p/SOX9 axis

Finally, we determined whether H19 promotes the PL‐induced chondrogenic differentiation of hUCMSCs by regulating the miR‐29b‐3p/SOX9 axis. To this purpose, we coinfected hUCMSCs with Lenti‐NC/Lenti‐H19 and Lenti‐miR‐NC/Lenti‐miR‐29b‐3p, and then cultured them in 5% PL‐supplemented chondrogenic differentiation medium for 21 days. The results of the qRT‐PCR analysis showed that H19 expression was increased postinfection with Lenti‐H19 but decreased postinfection with Lenti‐miR‐29b‐3p (Fig. S1B). Alcian blue staining revealed that Lenti‐miR‐29b‐3p infection reduced production of proteoglycan in the differentiated cells and attenuated the H19 overexpression‐mediated promotion of proteoglycan content (Fig. 4C). Furthermore, the up‐regulation of protein levels of SOX9, collagen II and aggrecan in the H19‐overexpressing cells was rescued following Lenti‐miR‐29b‐3p infection (Fig. 4D). Collectively, these observations suggested that H19 promoted PL‐induced chondrogenic differentiation of hUCMSCs by regulating the miR‐29b‐3p/SOX9 axis.

## Discussion

MSCs are multipotent stem cells with the potential to differentiate into chondrocytes, and MSC‐derived cartilage tissue engineering is a clinical method used for OA treatment [23]. PRP is a blood extraction product widely used in clinical practice to accelerate the wound healing process of some tissues, such as muscle [6], cartilage [7] and tendon [8]. Prepared from PRP, PL has been shown to reduce immunogenicity [10] and promote MSC chondrogenic differentiation, providing broad application prospects for allogeneic or xenogeneic transplantation in cartilage‐related diseases [24, 25]. A previous study clearly showed that PL induced the differentiation of hUCMSCs into chondrocytes [12]. In this study, we demonstrated for the first time the molecular mechanism by which PL induces MSC chondrogenic differentiation; that is, PL induced chondrogenic differentiation of hUCMSCs by regulating the H19/miR‐29b‐3p/SOX9 axis.

A number of recent studies have suggested that lncRNA plays a critical role in chondrogenic differentiation of MSCs. An increasing number of lncRNAs have been identified with a potential role in chondrogenesis, such as ROCR [14], differentiation antagonizing non‐protein‐coding RNA [26], maternally expressed 3 [23] and ADAMTS9 antisense RNA 2 [27]. For instance, lncRNA urothelial cancer‐associated 1 (UCA1) promotes chondrogenic differentiation of human bone marrow‐derived MSCs via regulating the miR‐145‐5p/SMAD5 and miR‐124‐3p/SMAD4 axis [28]. lncRNA H19 has been shown to promote the osteogenic differentiation of MSCs [15, 16] and inhibit the differentiation MSCs into adipocytes [17]. Yang *et al*. [18] demonstrated that in the bovine mammary alveolar cell–T cell line, TGF‐β induced up‐regulation of H19, which promoted epithelial‐to‐mesenchymal transition and contributed to mammary gland fibrosis. PL contains a relatively large number of growth factors, including TGF‐β that is critical for chondrogenic differentiation [10]. Thus, we speculated that H19 might be involved in the PL‐induced chondrogenic differentiation of hUCMSCs. To our knowledge, our findings in this study provided the first evidence that H19 overexpression promoted, whereas H19 silencing attenuated the chondrogenic differentiation of PL‐treated MSCs. Evidence indicates that TGF‐β1 up‐regulated H19 expression through activating the phosphoinositide‐3‐kinase/AKT pathway in epithelial cells [29]. Whether the phosphoinositide‐3‐kinase/Akt pathway or other transcriptional regulation mechanisms are involved in the PL‐induced H19 up‐regulation remains to be further investigated.

Several studies have established that H19 regulates the expression and biological functions of certain miRNAs by acting as a competing endogenous RNA or a miRNA sponge [30, 31]. For example, H19 contributes to oxidative damage repair in the early age‐related cataract by sponging miR‐29a [32]. miR‐29b‐3p not only plays an important role in tumor initiation and progression [33] but also participates in DNA damage response [34], tissue fibrosis [35] and chondrogenic differentiation [36]. In this study, we performed bioinformatics analysis and luciferase reporter assay to analyze the interaction between miR‐29b‐3p and H19 or SOX9. Results showed that H19 can function as a sponge RNA for miR‐29b‐3p that negatively regulates the expression of target gene *SOX9*. *SOX9* gene is a cartilage‐specific transcription factor that controls the expression of numerous chondrocyte genes (e.g. collagen II and aggrecan) and plays essential roles in chondrocyte differentiation and cartilage formation [37, 38]. Our results demonstrated that H19 positively regulated SOX9 expression and promoted MSC chondrogenic differentiation, and these effects were rescued by miR‐29b‐3p mimic.

## Conclusion

The findings in this study demonstrate that H19 expression is induced in PL‐stimulated hUCMSCs and that H19 acts as a sponge of miR‐29b‐3p to up‐regulate the expression of the master chondrocyte transcription factor SOX9, thereby promoting PL‐induced chondrogenic differentiation of hUCMSCs. These data provide new experimental evidence for elucidating the mechanism of MSC in cartilage repair and a novel therapeutic option for OA.

## Conflict of interest

The authors declare no conflict of interest.

## Author contributions

BC designed the study. BC and XD participated in the experiments. BC and XD contributed to the data analysis. BC drafted the paper. All authors approved the paper.

## Supporting information


**Fig. S1.** Expression of H19 in hUCMSCs postinfection with the indicated lentiviruses. (A) qRT‐PCR analysis of H19 expression in hUCMSCs infected with Lenti‐NC, Lenti‐H19, Lenti‐shRNA and Lenti‐sh‐H19 after culture in 5% PL‐supplemented chondrogenic differentiation medium for 21 days. **P < 0.01, vs. Lenti‐NC; ^##^ *P* < 0.01, vs. Lenti‐shRNA (Student’s *t*‐test). (B) qRT‐PCR analysis of H19 expression in hUCMSCs coinfected with Lenti‐NC/Lenti‐H19 and Lenti‐miR‐NC/Lenti‐miR‐29b‐3p after culture in 5% PL‐supplemented chondrogenic differentiation medium for 21 days. **P < 0.01, vs. Lenti‐miR‐NC + Lenti‐NC; ^#^ *P* < 0.05, vs. Lenti‐miR‐NC + Lenti‐H19 (ANOVA). The quantitative statistics were presented as the mean ± standard deviation (*n* = 3).

## Data Availability

The data that support the findings of this study are available from the corresponding author upon reasonable request.
