# Supplementary figures and images for: Platelet lysate induces chondrogenic differentiation of umbilical cord‐derived mesenchymal stem cells by regulating the lncRNA H19/miR‐29b‐3p/SOX9 axis
Source: FEBS Open Bio. 2020 Nov 6;10(12):2656–65. doi: 10.1002/2211-5463.13002 (PMC7714074; doi:10.1002/2211-5463.13002)

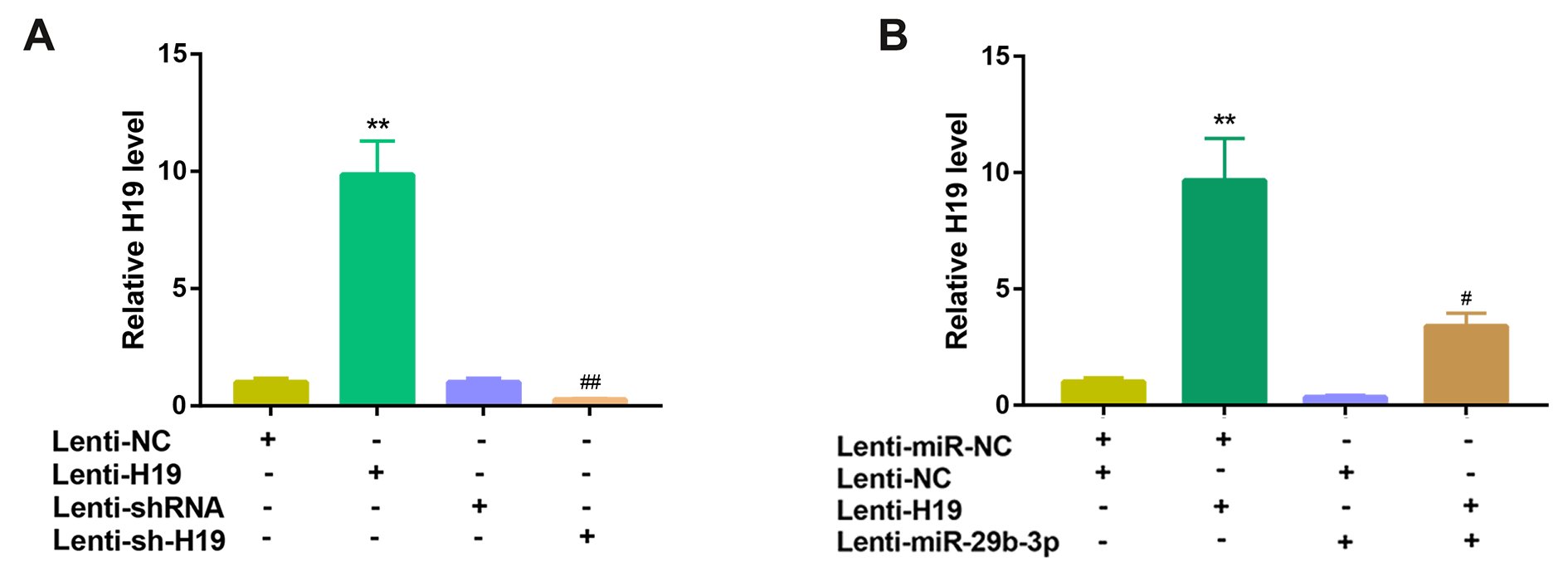

Supplement: Supplementary file 1 — Fig. S1. Expression of H19 in hUCMSCs postinfection with the indicated lentiviruses. (A) qRT‐PCR analysis of H19 expression in hUCMSCs infected with Lenti‐NC, Lenti‐H19, Lenti‐shRNA and Lenti‐sh‐H19 after culture in 5% PL‐supplemented chondrogenic differentiation medium for 21 days. **P < 0.01, vs. Lenti‐NC; ## P < 0.01, vs. Lenti‐shRNA (Student’s t‐test). (B) qRT‐PCR analysis of H19 expression in hUCMSCs coinfected with Lenti‐NC/Lenti‐H19 and Lenti‐miR‐NC/Lenti‐miR‐29b‐3p after culture in 5% PL‐supplemented chondrogenic differentiation medium for 21 days. **P < 0.01, vs. Lenti‐miR‐NC + Lenti‐NC; # P < 0.05, vs. Lenti‐miR‐NC + Lenti‐H19 (ANOVA). The quantitative statistics were presented as the mean ± standard deviation (n = 3). [file FEB4-10-2656-s001.tif]
